# Surgical outcome of clipping in anterior circulation aneurysms

**DOI:** 10.12669/pjms.40.12(PINS).11119

**Published:** 2024-12

**Authors:** Talha Sajid, Arooj Kiran, Adeel-Ur-Rehman, Ismaeel Khalid, Zubair Khan, Abdul Majid

**Affiliations:** 1 Dr. Talha Sajid, MBBS, Resident Neurosurgery, Department of Neurosurgery, Punjab Institute of Neurosciences, Lahore, Pakistan; 2 Dr. Arooj Kiran, MBBS, Resident Neurosurgery, Department of Neurosurgery, Punjab Institute of Neurosciences, Lahore, Pakistan; 3 Dr. Adeel-Ur-Rehman, MBBS, Resident Neurosurgery, Department of Neurosurgery, Punjab Institute of Neurosciences, Lahore, Pakistan; 4 Dr Ismaeel Khalid Iqbal, MBBS, Resident Neurosurgery, Department of Neurosurgery, Punjab Institute of Neurosciences, Lahore, Pakistan; 5 Dr. Zubair Ahmed Khan, MBBS, FCPS, Senior Registrar Neurosurgery, Department of Neurosurgery, Punjab Institute of Neurosciences, Lahore, Pakistan; 6 Prof. Dr. Abdul Majid, MBBS, FCPS, MCPS, Head Department of Neurosurgery, Department of Neurosurgery, Punjab Institute of Neurosciences, Lahore, Pakistan

**Keywords:** Ruptured Intracranial Aneurysm, Subarachnoid Hemorrhage, Vasospasm, Hydrocephalus, Ventriculoperitoneal Shunt

## Abstract

**Objective:**

To observe the outcome of surgical clipping in anterior circulation aneurysm in a modestly resourced hospital.

**Methods:**

A retrospective cross-sectional study was conducted at Punjab Institute of Neurosciences Lahore, from August 2022 to July 2023. Seventy five patients meeting the inclusion criteria of age <65, saccular aneurysm of anterior circulation, and Hunt and Hess grade one or two were enrolled through non-probability convenience sampling. Data were collected from patient records, and surgeries were performed by experienced neurosurgeons using pterional and sub-frontal approaches. SPSS version 26 was utilized for data analysis.

**Results:**

Mean age 49.3733 ± 2.56 (Mean ± SD) years, 60% female. Most common aneurysm location: anterior communicating artery (37.33%). Post-operative complications: vasospasm (17.33%), hydrocephalus (6.66%), fits (5.33%), mortality (2.66%). About 91% patients had no complications or recovered within three months.

**Conclusion:**

Surgical clipping of anterior circulation aneurysms is a safe and effective treatment, yielding favorable angiographic outcomes. Despite occasional complications, most patients achieved satisfactory results, especially in a setting having limited endovascular facilities or when coiling is not feasible. Future comparative studies with endovascular methods will further refine patient selection and surgical techniques.

## INTRODUCTION

An aneurysm is an outpouching of an artery. Intracranial aneurysms occur more frequently than extracranial ones, primarily because intracranial arteries lack an external elastic lamina and exhibit discontinuity of the tunica media at the points were smaller vessels branch from larger parent vessels. Additionally, congenital factors such as degenerative changes, hypertension, atherosclerosis, connective tissue disorders, and hemodynamic stress significantly contribute to the development of intracranial aneurysms. The rupture of these aneurysms leads to substantial morbidity and mortality, predominantly located at arterial bifurcations. The major clinical manifestation of aneurysm rupture is subarachnoid hemorrhage, which can result in numerous complications, both intracranial and extracranial. Managing these aneurysms poses considerable challenges.

Various studies have aimed to evaluate and compare different treatment options and their outcomes, yet no definitive conclusion or gold standard treatment has been established. For example, a study by Park et al. demonstrated the safety, feasibility, and efficiency of surgical clipping for anterior circulation aneurysms.^1^ Similarly, research by Mohammad et al., comparing the safety of clipping versus coiling, concluded that both techniques are safe, each with its own advantages and disadvantages, but no sufficient data exists to definitively prefer one over the other.^2^ Emphasizing the importance of comprehensive knowledge in anatomy, physiology, topography, and pathology, Lucifero et al. highlighted the necessity of understanding the vasculature of the brain, head, and neck for any significant benefit from clipping or coiling interventions.^3^ A 2023 meta-analysis by Fotakopoulos et al., involving 18 studies and a total of 3,353 patients with unruptured intracranial aneurysms, suggested better surgical outcomes in patients with anterior circulation aneurysms compared to those with posterior circulation aneurysms. However, since most aneurysms in our context present as ruptured, the results of this analysis cannot be considered a standard.^4^ Another meta-analysis by Peng et al. found coiling to be significantly associated with improved quality of life, though it exhibited a higher rate of mortality, rebleeding, and hydrocephalus compared to clipping.^5^

The objective of our study was to analyze the outcomes of clipping and its complications in patients with anterior circulation aneurysms in developing countries, such as Pakistan. We aimed to evaluate these complications in terms of better outcomes, morbidity, and mortality.

## METHODS

A retrospective cross-sectional study was conducted at the Punjab Institute of Neurosciences, Lahore. A total of 75 patients were enrolled in the study through non-probability convenience sampling, meeting the predefined inclusion criteria.

### Ethical Approval:

It was taken from the institutional IRB Ref. No: 1782/IRB/PINS/approval/2024.

### Inclusion criteria:

• The study included patients of any gender, younger than 65 years, with saccular aneurysms of the anterior circulation, presenting more than 14 days post-bleed, and classified as Hunt and Hess Grade-1 or 2.

### Exclusion criteria:

• Patients older than 65 years, those with fusiform aneurysms or aneurysms of the posterior circulation, presenting with Hunt and Hess Grade-3 or higher, were excluded. Additionally, patients deemed unfit for general anesthesia or any surgical intervention for any reason were also excluded.

### Data collection:

Data were collected by using Google Forms from the hospital’s patient records, including patient files, Medical Registration Numbers, and the PACS online system, after obtaining permission from the department head. Enrollment was limited to patients who met the inclusion criteria. A team of neurosurgeons with extensive experience conducted the surgeries and managed post-procedure follow-ups according to the departmental protocol for three months. The surgical approach was either pterional or sub-frontal. Comprehensive information including name, age, gender, address, pre-operative and post-operative CT scans, baseline data, Hunt and Hess grade, surgeon’s name, post-operative hospital stay, and post-operative complications was meticulously recorded by resident neurosurgeons. The study’s exclusion criteria were strictly adhered to, minimizing bias and confounders.

### Data analysis:

The collected data were analyzed using a Microsoft Excel sheet. Continuous variables, such as age, were summarized with means and standard deviations, while categorical variables, including gender and location of the aneurysm, were presented as frequencies. Post-operative complications were depicted in bar graphs and frequencies.

## RESULTS

### Gender distribution and age:

In our study, 60% (45) of the participants were female, while 40% (30) were male. The mean age of the patients was 49.3733 ±2.56 (Mean ± SD) years.

### Location of aneurysm:

The most common location of an aneurysm among the patients was in the anterior communicating artery (37.33%), while the least common was in the peri-callosal artery (4%). This trend was similar across both genders. Details are presented in Table-I.

**Table-I:** Demographic characteristics of the anterior circulation aneurysm.

Location	Females Frequency (%)	Males Frequency (%)	Total Frequency (%)
Anterior Communicating Artery Aneurysm	16 (21.3%)	12 (16%)	28 (37.33%)
Middle Cerebral Artery Aneurysm	13 (17.33%)	09 (12%)	22 (29.33%)
Posterior communicating Artery Aneurysm	07 (9.33%)	05 (6.66%)	12 (16%)
Internal Carotid Artery Bifurcation Aneurysm	07 (9.33%)	03 (4%)	10 (13.33%)
Peri-callosal Artery Aneurysm	02 (2.66%)	01 (1.33%)	03 (4%)
	45 (60%)	30 (40%)	75 (100%)

### Post-Operative Complications:

**Fig.1:**
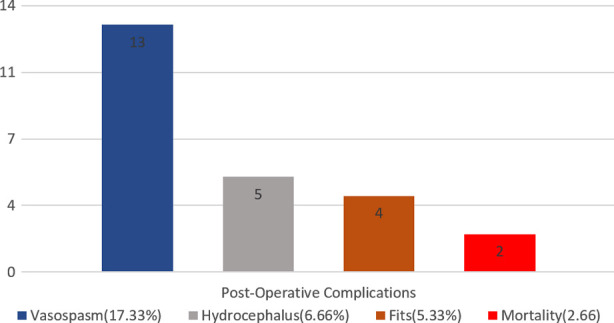
Post-operative complications following clipping of anterior circulation aneurysm.

A thorough follow-up revealed the presence of the following complications in the immediate post-operative period: vasospasm was the most common complication (17.33%), followed by hydrocephalus (6.66%). Fits were observed in 5.33% of the patients, and the mortality rate was 2.66%, as illustrated in Fig.1.

Among the 13 cases who developed vasospasm, neurological deficits occurred in 38.4%, and a 15.38% mortality rate was observed. In 46.15% of the cases, vasospasm resolved without any further complications. Of the five cases that developed hydrocephalus post-operatively, 60% were managed conservatively, while 40% required further surgery in the form of ventriculoperitoneal (VP) shunting.

### Outcome of the study:

The results of this study were quite favorable, indicating that 91% of the patients experienced no complications or recovered from complications without any long-term deficits within the three-month follow-up period. New neurological deficits developed in 6.66% of patients, and 2.66% of patients died post-operatively.

## DISCUSSION

Recent advancements in endovascular interventions, such as detachable coils and flow diversion, have revolutionized the treatment of brain aneurysms, making these methods more preferred over traditional surgery. However, a study by Taha MM et al., in 2019, stated that in countries with limited resources, these procedures can be prohibitively expensive and beyond the reach of the majority of the population. As a result, in our center, traditional surgery, specifically surgical clipping, is still commonly used to treat ruptured aneurysms.^6^

Our study supports the effectiveness of clipping for anterior circulation aneurysms, in contrast to a study by Wadd et al. conducted at Lahore General Hospital from January 2010 to December 2013, which compared clipping versus coiling in anterior circulation aneurysms and found a 68.6% favorable outcome after one year in patients who underwent clipping with Hunt and Hess Grades-1 and 2, as compared to an 80% favorable outcome in the coiling group. Surgical treatment was associated with poorer outcomes and a relatively higher re-bleeding risk, while endovascular treatment presented lower risks of seizure, delayed cerebral ischemia, ischemic lesions on MRI, and in-hospital complications. Early neurological deterioration was more common in elderly patients with subarachnoid hemorrhage (SAH), partly due to the SAH and partly due to procedure-related factors. Complications, including infection and epilepsy, affected 5% of patients, with hydrocephalus being more common in the elderly. Shunt implantation was less frequent, possibly due to younger patient age and better SAH grades.^7^

The International Subarachnoid Aneurysm Trial (ISAT) and Kuopio studies demonstrated comparable outcomes between coiling and clipping, with ISAT indicating better outcomes for coiling. However, endovascular coiling was associated with better outcomes but carried higher risks of aneurysm recurrence and re-bleeding. The observed improvement in clipping outcomes over the last decade could be attributed to enhanced microsurgical techniques and expertise.^7^-^9^ In our study, only 13 cases developed vasospasm, and almost half of these patients recovered without any further deficit or complication. However, with advancements in clipping techniques, these complications could be further reduced.

Currently, some centers are employing the keyhole approach for clipping, unlike in our study where we used sub-frontal or pterional approaches. A study by Shao D et al., 2021 shows that the keyhole approach leverages the normal intracranial anatomical space to minimize brain retraction, thereby reducing operative damage, postoperative complications (such as epilepsy and bleeding), the amount of blood needed for the operation, and the cost, while facilitating quicker postoperative recovery, a milder postoperative response, and favorable operative outcomes. Nonetheless, the keyhole approach has certain limitations and requires surgeons to possess specific skills to manage emergency complications during and after surgery. Studies like ISAT suggest that while endovascular therapy may have higher recurrence and re-bleeding rates, surgical clipping remains preferable in certain scenarios.^10^,^11^

Recent studies, including one reviewed by Whitfield and Kirkpatrick in 2001, have shown that operating earlier, within the first three days following the hemorrhage, leads to better outcomes compared to delayed surgery. Maurice-Williams and Lafuente even suggested that the timing of surgery has minimal effect on overall management outcomes (Maurice-Williams et al., 2003). However, in our study, clipping was performed on anterior circulation aneurysms with Hunt and Hess Grades-1 and 2, after 14 days of hemorrhage (SAH). Despite symptomatic vasospasm remaining a leading cause of delayed morbidity and mortality, microsurgical clipping techniques facilitate safe treatment. Therefore, further studies are needed to demonstrate this effect in our center.^12^

Certain risk factors are associated with poorer outcomes following surgical clipping, such as smoking and the use of temporary clips. A 13 years study by Sharma GR et al. analyzed 500 patients with ruptured intracranial aneurysms, identifying 28 cases (5.6%) of ruptured distal anterior cerebral artery (DACA) aneurysms. Among these, 71.4% of patients had low-grade and 28.6% had high-grade Hunt and Hess (H&H) scores. At discharge, 67.8% showed good recovery, 21.5% were severely disabled, and 10.7% had died. Smoking and the use of temporary clips were significant predictors of adverse outcomes (p-values 0.03 and 0.00, respectively) at discharge and at the last follow-up. Despite historically poorer outcomes for DACA aneurysms, excellent overall outcomes were achieved with microsurgical clipping. Alcohol consumption and temporary clip usage were predictors of poor outcomes at discharge, while smoking and temporary clip usage were risk factors at the last follow-up. The limiting factor of this study includes its small sample size, but it underscores the importance of smoking cessation and cautious use of temporary clips in improving post-clipping outcomes.^13^

In our study, the postoperative mortality rate was only 2.66%, whereas mortality was higher with coiling. A study by Lindgren AE et al. observed that coiling had a higher risk of death within 14 days compared to clipping. This pattern persisted at the 90-day post-surgery mark, with a 14-day case-fatality rate for coiling versus clipping of 1.32 (95% CI 1.10-1.58). The clinical dataset’s crude 14-day case fatality rate was 5.7% (95% CI 4.2%-7.8%) for clipping and 9.0% (95% CI 7.3%-11.2%) for coiling. In multivariable logistic regression analysis, the odds ratio (OR) for 14-day case-fatality after coiling compared to clipping was 1.7 (95% CI 1.1-2.7), and for 90-day case-fatality, it was 1.28 (95% CI 0.91-1.82), with a 90-day poor functional outcome OR of 0.78 (95% CI 0.6-1.01). Optimal timing for performing microsurgical clipping after an aneurysmal subarachnoid hemorrhage has been debated for many years.^14^

A study by Muhammad F et al. on 200 patients who were diagnosed with unruptured anterior circulation aneurysms shows that durability was greater in the clipping group than in the coiling group. Although the conclusive outcome was almost even between treatments, the ratio of the ischemic events was higher in the coiling group than the clipping group. In contrast, the risks of specific complications involved in surgical procedure led to the extent of the postoperative hospital period following clipping.^15^

One study indicated that surgical clipping is a safe and reliable treatment and can be the first choice for ruptured aneurysms of the anterior circulation with a good grade, showing favorable outcomes in 91% of a total of 75 patients, while 9% experienced poor outcomes in terms of morbidity and mortality. Our study further supports these conclusions, suggesting that clipping can be a first-line treatment in centers with modest resources.

### Limitations:

The limitations of our study include its retrospective nature, small patient sample size, focus solely on surgical outcomes rather than overall patient management, and evaluation of patients with delayed cerebral ischemic deficits using brain CT rather than diffusion MRI. Nonetheless, it concludes that clipping is safe in developing countries, despite some logistic difficulties and economic burdens. This study demonstrates that surgical clipping remains a viable and favorable treatment method for anterior circulation aneurysms.

## CONCLUSION

Surgical clipping of anterior circulation aneurysms represents a viable and effective treatment option particularly in scenarios where coiling may not be feasible or suitable, or in centers limited endovascular facilities. While surgical complications were observed in a subset of cases, the majority of patients achieved satisfactory clinical results. Further research is needed to refine patient selection criteria and surgical techniques to optimize outcomes in this patient population. To enhance the presentation of the outcomes of the current study, a comparative analysis with endovascular methods is planned once the data collection is complete.

### Authors Contribution:

**TS** conceived, designed and did statistical analysis, editing of manuscript.

**TS, AK and IK** collected the data.

**TS, AK and AR** wrote the manuscript.

**ZK** did review and final approval.

**ZK and AM** critically reviewed and supervised the study.

All authors have read the final manuscript and are responsible for integrity of the study.
